# Increased Resting-State Interhemispheric Functional Connectivity of Posterior Superior Temporal Gyrus and Posterior Cingulate Cortex in Congenital Amusia

**DOI:** 10.3389/fnins.2021.653325

**Published:** 2021-04-30

**Authors:** Zhishuai Jin, Sizhu Huyang, Lichen Jiang, Yajun Yan, Ming Xu, Jinyu Wang, Qixiong Li, Daxing Wu

**Affiliations:** ^1^Medical Psychological Center, The Second Xiangya Hospital, Central South University, Changsha, China; ^2^Medical Psychological Institute, Central South University, Changsha, China; ^3^National Clinical Research Center for Mental Disorders, Changsha, China

**Keywords:** congenital amusia, functional connectivity, voxel-mirrored homotopic connectivity, posterior superior temporal gyrus, posterior cingulate cortex

## Abstract

Interhemispheric connectivity of the two cerebral hemispheres is crucial for a broad repertoire of cognitive functions including music and language. Congenital amusia has been reported as a neurodevelopment disorder characterized by impaired music perception and production. However, little is known about the characteristics of the interhemispheric functional connectivity (FC) in amusia. In the present study, we used a newly developed voxel-mirrored homotopic connectivity (VMHC) method to investigate the interhemispheric FC of the whole brain in amusia at resting-state. Thirty amusics and 29 matched participants underwent a resting-state functional magnetic resonance imaging (fMRI) scanning. An automated VMHC approach was used to analyze the fMRI data. Compared to the control group, amusics showed increased VMHC within the posterior part of the default mode network (DMN) mainly in the posterior superior temporal gyrus (pSTG) and posterior cingulate cortex (PCC). Correlation analyses revealed negative correlations between the VMHC value in pSTG/PCC and the music perception ability among amusics. Further ROC analyses showed that the VMHC value of pSTG/PCC showed a good sensibility/specificity to differentiate the amusics from the controls. These findings provide a new perspective for understanding the neural basis of congenital amusia and imply the immature state of DMN may be a credible neural marker of amusia.

## Introduction

Interhemispheric connectivity is a prominent feature of macroscopic brain organization characterized by a high level of collaboration between bilateral brain regions (Serrien et al., 2006). Proper integration of the two cerebral hemispheres is crucial for a broad repertoire of cognitive functions including music and language (Gazzaniga, 1995; Gotts et al., 2013; Rosenthal, 2016). Conversely, disturbances of hemispheric coordination may impact cognitive and behavioral functioning (Gazzaniga, 1995; Toga and Thompson, 2003). For example, abnormal hemispheric integration has been associated with developmental disorders, such as dyslexia, autism spectrum disorder (Anderson et al., 2011; Richlan et al., 2011).

Congenital amusia is a neurodevelopmental disorder, which has been characterized by impaired music perception and production by several seminal studies (Ayotte et al., 2002; Foxton et al., 2004; Peretz et al., 2002). The dominant theory of the neural basis of amusia focused on the superior temporal gyrus (STG), inferior frontal gyrus (IFG), and the connectivity between them in structure and function in the right hemisphere (Hyde et al., 2006, 2007; Loui et al., 2009; Albouy et al., 2013; Peretz, 2016). However, multiple lines of evidence suggest that amusia may involve abnormalities in interhemispheric connectivity and cooperation. The early studies of amusia have reported music perception probably involved both hemispheres and activated cross-hemisphere neural substrates of melodic and temporal information processing in the human brain (Peretz, 1985; Estanol and Mendez, 1998; Schuppert et al., 2000; Schurz et al., 2015). Tract-based spatial statistics (TBSS) studies also found both congenital amusia (Wang et al., 2017) and acquired amusia (Sihvonen et al., 2017) had abnormal white matter tracts including the corpus callosum (CC), which is one component of the commissural system that mediates interhemispheric interaction (Hoptman and Davidson, 1994). Furthermore, MEG studies found the abnormal melody encoding responses of amusia are related to the increased connectivity of bilateral auditory cortexes (Albouy et al., 2013, 2015), as well as enhanced connectivity in task fMRI of passively listening to sequences (Hyde et al., 2011) and resting-state fMRI (Leveque et al., 2016) excepting reduced right frontotemporal connectivity. Therefore, an important open question regarding interhemispheric connectivity in amusia remains which can improve our understanding of the neural basis of amusia.

Notably, since the first resting-state fMRI study of Biswal et al. (1995), it has provided powerful insights into functional anomalies of various psychiatric disorders (Hoptman et al., 2012; Guo et al., 2013, 2014), as well as neurodevelopment disorders such as amusia, prosopagnosia, and dyslexia (Schurz et al., 2015; Leveque et al., 2016; Zhao et al., 2018). One prominent property of hemispheric functional organization in terms of resting-state functional connectivity is homotopy, which is the synchronicity in spontaneous activity of interhemispheric between corresponding regions (Salvador et al., 2005; Stark et al., 2008). Recently, a new method (voxel-mirrored homotopic connectivity, VMHC) has been proposed as a validated approach to quantify the resting-state functional connectivity between each voxel in one hemisphere and its corresponding voxel in the opposite hemisphere (Zuo et al., 2010). This approach differs from analyses of classic functional connectivity, which requires *a priori* selection of a voxel, cluster, or anatomical brain atlas region (Friston, 2011). It explored voxel-mirrored functional connectivity in symmetrical whole brain space based voxel. Besides, the VMHC index emphasizes the feature that specific patterns of interhemispheric connectivity can reflect functional consequences of interhemispheric communication in neurodegenerative, development, or psychiatric disorders (Zuo et al., 2010). Abnormal VMHC patterns in widespread brain regions have been reported on depression (Hermesdorf et al., 2016; Fan et al., 2018), schizophrenia (Hoptman et al., 2012; Guo et al., 2018), autism (Kozhemiako et al., 2019), mild cognitive impairment (MCI) (Luo et al., 2018), and conduct disorder (Lu et al., 2020), which indicated that VMHC is a reliable neural marker for brain function.

Given the prior findings summarized above, it is little known about the character of the interhemispheric functional connectivity (FC) and whether the character can be a neural marker of amusia, which may help understand the neural basis of congenital amusia. Accordingly, the first aim of the present study was to test the hypothesis that individuals with amusia may have abnormal interhemispheric FC in several different functional brain regions compared to the control group by the VMHC. Secondly, we tested the hypothesis that the abnormal interhemispheric FC was associated with the music perception ability by correlation analyses of the VMHC value with the musical test. Thirdly, we tested the hypothesis that whether the VNHC value of potential abnormal clusters in amusia can be credible neural markers of amusia evaluated by Receiver Operator Characteristic (ROC) curves.

## Materials and Methods

### Participants

Native Mandarin-speaking participants were recruited from advertisements and campus screening. All participants reported right-handed, with no professional musical training, and no neurological or audiological deficit. All participants signed the informed consent form, and the study design was approved by the ethics committee of the Second Xiangya Hospital of Central South University. All participants could withdraw from the study if they experienced discomfort.

### Amusia Identification

All participants (age range: 18–24) were confirmed to have normal binaural hearing by pure tone audiometry. Amusia was defined by the Montreal Battery Evaluation of Amusia (MBEA). The MBEA includes three melodic subtests based on the pitch (scale, contour, and interval), two temporal subtests based on the time (rhythm and meter), and one musical memory subtest, and has been an effective tool to screen for amusia in western and eastern cultures (Peretz et al., 2003; Nan et al., 2010). According to the previous study of amusia in Chinese college students, individuals with a global music score (MBEA average) less than 21.5 were defined as amusia (Nan et al., 2010). The amusia and control groups were matched in age, gender, and years of education (see Table 1). Moreover, all participants conducted the Chinese-revised Wechsler Adult Intelligence Scale to rule out the influence of intelligence quotient (IQ) (Gong, 1983).

**TABLE 1:** Characteristics of study participants by group.

Characteristic	Amusia *n* = 30 M ± SD	Control *n* = 29 M ± SD	χ^2^/t	*p*
Male/Female	13/17	15/14	0.15	0.70
Age(year)	19.73 ± 2.07	19.00 ± 1.17	1.67	0.10
Education(year)	13.80 ± 1.38	13.48 ± 0.91	1.04	0.30
IQ	106.10 ± 9.55	106.48 ± 9.80	–0.15	0.88
FD	0.05 ± 0.02	0.06 ± 0.03	–1.59	0.12
Global music score	19.57 ± 1.73	27.13 ± 0.97	–20.81	<0.001
Melodic subtest	19.34 ± 2.04	27.80 ± 0.92	–20.64	<0.001
Temporal subtest	19.26 ± 2.46	25.64 ± 2.00	–10.91	<0.001
Memory subtest	20.87 ± 3.57	28.10 ± 1.82	–9.84	<0.001

*Categorical data of gender was tested using chi squared test (χ^2^); variables such as age, education, intelligence quotient (IQ), and framewise displacement (FD) were tested by two-sample *t*-test (indicated by *t*); M and SD indicate the mean and the standard deviation. Global music score was the average of scale, contour, and interval, rhythm, meter, and memory; Melodic subtest is the score averaged over scale, contour and interval; Temporal subtest is the score averaged over rhythm and meter; Memory subtest is the score of music memory.*

### Image Acquisition

Magnetic resonance imaging scans were acquired using a 3T Siemens Skyra scanner (Siemens, Erlangen, Germany). All participants were instructed to lie in a comfortable position, with eyes closed, and remaining awake. Resting-state functional magnetic resonance images (fMRI) time series were acquired with an echo-planar imaging sequence with the following parameters: repetition time, 2000 ms; echo time, 30 ms; flip angle, 80°; voxel size, 4.0 mm × 4.0 mm × 4.0 mm; the field of view, 256 mm × 256 mm; consisting of 32 slices covering the whole brain, and 216 volumes were obtained per participant. Simultaneously, high- resolution three-dimensional T1-weighted images were acquired using a gradient-echo sequence with the following parameters: repetition time, 1900 ms; echo time, 2.03 ms; flip angle, 9°; voxel size, 1.0 mm × 1.0 mm × 1.0 mm; slice thickness, 1 mm, the field of view, 256 mm × 256 mm; the number of slices, 176. All images were checked by a radiologist, who reported no anomalous findings.

### Data Preprocessing

The DPABI v4.5 software was applied to preprocess the MRI images^1^ (Yan et al., 2016). Firstly, removed the first 10 time points of each participant to ensure the stability of MRI signals, the rest 206 volumes of the functional images were slice-timing corrected for temporal differences and realigned for head movement correction. No participants had a motion more than 2 mm of translation or 2° of rotation. Secondly, each participant’s T1 images were segmented into white matter, gray matter, and cerebrospinal fluid in the standard Montreal Neurological Institute (MNI) space by using the “New Segment + DARTEL” strategy to create a study-specific template. Each participant’s functional scans were spatially normalized to MNI space using the study-specific template and resampled to 3 mm× 3 mm× 3 mm voxels. Then the normalized functional images were regressed out several nuisance covariates including cerebrospinal fluid, white matter signals, as well as Friston-24 head motion parameters. Finally, the functional images were smoothed with a 6-mm full width at half maximum (FWHM) isotropic Gaussian kernel to improve the signal-to-noise ratio, detrended linear trend, and filtered at 0.01–0.1 Hz to reduce the physiological noises.

### Voxel-Mirrored Homotopic Connectivity Computation

To minimize the geometric differences between the left and the right hemispheres, we refined the registration from individual anatomical to MNI152 template space. Firstly, all 59 normalized T1 images were averaged to create a mean normalized T1 image. This image was then averaged with its left-right mirrored version to generate a group-specific symmetrical template. Secondly, each participant’s normalized T1 images were registered to the symmetric template and applied the registration parameter to each participant’s preprocessed functional data. Finally, the homotopic connectivity coefficient of each participant was calculated by Pearson’s correlation coefficient between the time series of each pair of mirrored interhemispheric voxels. Correlation values were then Fisher Z-transformed, which were referred to as the VMHC (Zuo et al., 2010; Lu et al., 2020; Zhao et al., 2020).

### Statistic Analyses

Chi-square test was used to compare gender, and two-sample *t*-tests were used to compare age, gender, IQ, framewise displacement (FD), and MBEA scores (global musical score, melodic subtest, temporal subtest, memory subtest) between the amusia group and control group. FD values were calculated for each participant in data preprocessing (Power et al., 2012).

Voxel-based Two-sample *t*-tests were used to compare VMHC maps between the amusia group and control group in DPABI software. Age, gender, IQ, and mean FD were applied as covariates. The resulting statistical map was set at *p* < 0.001 and the significance level was set at *p* < 0.05 corrected by the threshold-free cluster enhancement (TFCE) method (Smith and Nichols, 2009; Chen et al., 2018).

To further examine potential core regions that could account for amusia, the mean VMHC values were extracted from these abnormal brain regions and conducted Pearson correlations with MBEA scores in all participants, amusia group, and control group. ROC analysis was conducted to examine whether the mean VMHC values of potential abnormal brain regions can be credible neural markers to identify the amusia from the controls using SPSS19.0 software. Notably, this method of ROC analysis has been used to assess whether the abnormal VMHC values obtained from group analysis could differentiate the disorder individuals from control individuals, such as conduct disorder (Lu et al., 2020), major depressive disorder (Guo et al., 2018), and schizophrenia (Liu et al., 2018).

## Results

### Characteristics of Participants

As seen in Table 1, there was no significant difference in gender (chi-squared test, *p* > 0.05) and no significant differences in age, years of education, intelligence quotient (IQ), and FD between the amusia and control group (*t*-tests, all *p* > 0.05). For the MBEA scores, amusia had a significantly lower global musical score (averaged over the six subtests of the MBEA) comparing to controls, as well as melodic subtest, temporal subtest, and memory subtest (*t*-tests, all *p* < 0.001).

### Group Differences in VMHC

**FIGURE 1:**
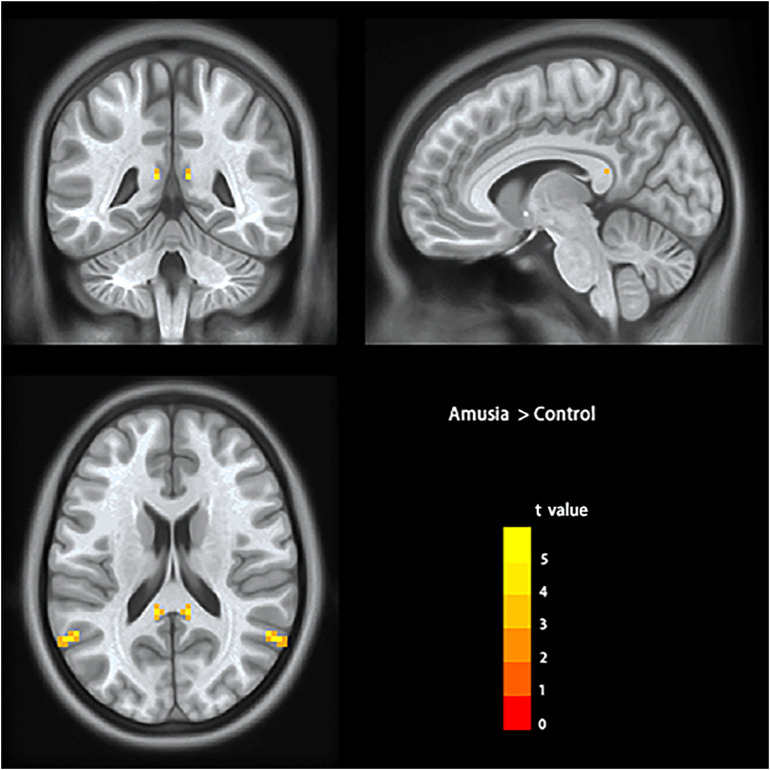
Voxel-mirrored homotopic connectivity results showed significantly increased homotopic functional connectivity (signal voxel significance was set at *p* < 0.001, TFCE corrected *p* < 0.05) in posterior superior temporal gyrus (pSTG) and posterior cingulate cortex (PCC) in amusics. The color bar indicates the *t* values of two-sample *t*-test analyses.

To test the hypothesis that whether there are abnormal interhemispheric FC of amusia, we conducted two-sample *t*-test analyses on the VMHC maps between amusia group and control group. Compared to control group participants, amusics showed significantly increased VMHC (*p* < 0.05, TFCE corrected) in the posterior superior temporal gyrus (pSTG: *x* = ± 60, *y* = –60, *z* = 21; voxel size 3 mm × 3 mm × 3 mm; 29 voxels), and posterior cingulate cortex (PCC: *x* = ± 9, *y* = –42, *z* = 15; voxel size 3 mm × 3 mm × 3 mm; six voxels) (Table 2 and Figure 1).

**TABLE 2:** Significant clusters on VMHC analysis.

Brain region	Cluster size (voxels)	MNI coordinates (*x, y, z*)	Peak *t* value
pSTG	29	(±60, –60, 21)	5.55
PCC	6	(±9, –42, 15)	5.51

*Signal voxel significance was set at *p* < 0.001 in VMHC comparison corrected by threshold-free cluster enhancement (TFCE, *p* < 0.05); VMHC, voxel-mirrored homotopic connectivity; MNI, Montreal Neurological Institute; pSTG, posterior superior temporal gyrus; PCC, posterior cingulate cortex; voxel size: 3 × 3 × 3mm^3^. The brain regions were reported according to the peak voxels in AAL based xjview (http://www.Alivelea~rn.net/xjview).*

### Correlation Analyses of MBEA Scores With the Interhemispheric Functional Connectivity

**FIGURE 2:**
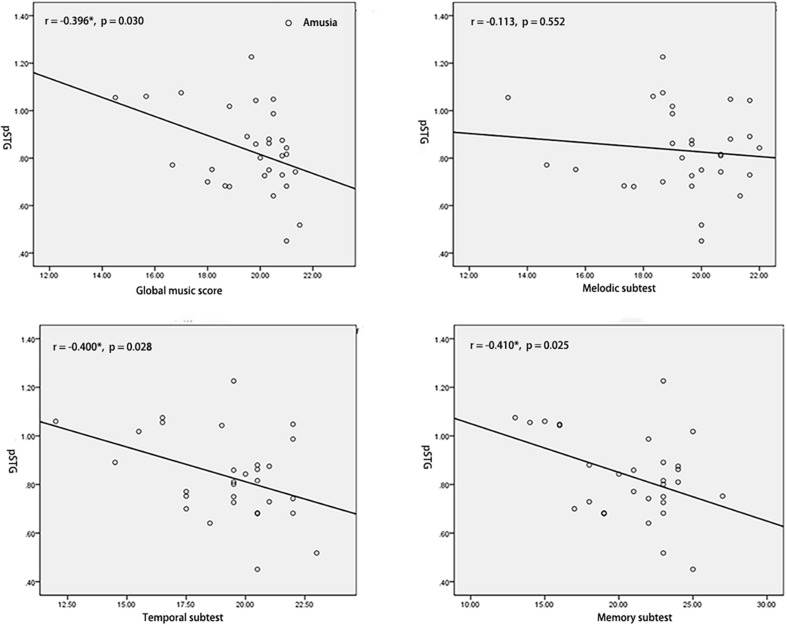
Correlation analyses between the mean value of pSTG and MBEA scores in amusia group. *p < 0.05.

**FIGURE 3:**
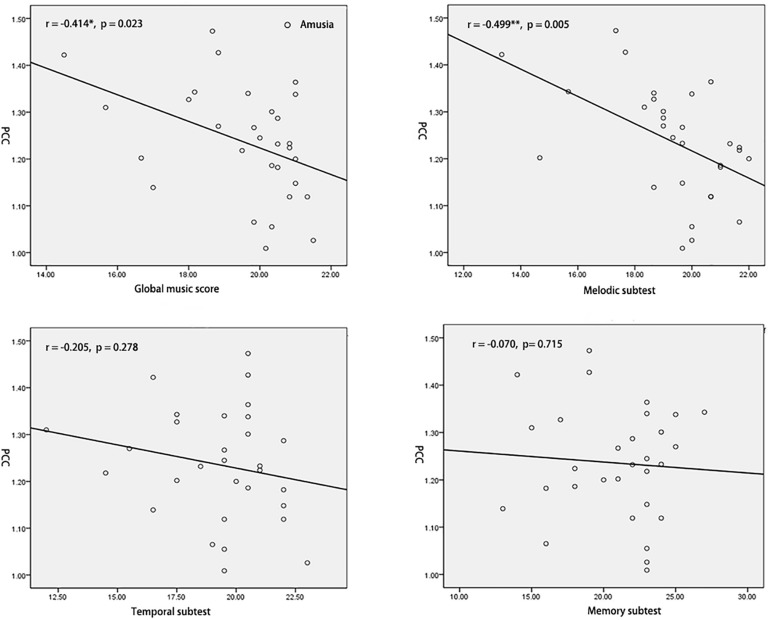
Correlation analyses between the mean value of PCC and MBEA scores in amusia group. *p < 0.05, **p < 0.01.

To test the hypothesis that whether the abnormal interhemispheric FC was associated with the music perception ability, we extracted the mean VMHC values of pSTG/PCC and conducted Pearson’s correlation analyses of pSTG/PCC with MBEA scores including global musical score, melodic subtest, temporal subtest, and memory subtest in all participants, amusia group, and control group. Among all participants, there were significant negative correlations of pSTG/PCC with MBEA scores (all *r* < –0.5, *p* < 0.001). Among amusia group, there were significant negative correlations of the pSTG with global musical score (*r* = –0.396, *p* = 0.030), temporal subtest (*r* = –0.400, *p* = 0.028), and memory subtest (*r* = –0.410, *p* = 0.025, Figure 2), as well as significant negative correlations of the PCC with global musical score (*r* = –0.414, *p* = 0.023), and melodic subtest (*r* = –0.499, *p* = 0.005, Figure 3). There was no significant correlation of the pSTG with melodic subtest (*r* = –0.113, *p* = 0.552), as well as no significant correlation of the PCC with temporal subtest (*r* = –0.205, *p* = 0.278) and memory subtest (*r* = –0.070, *p* = 0.715). Furthermore, there were no significant correlations between pSTG/PCC and MBEA scores among the control group (Supplementary Figures 1, 2).

### ROC Analysis

**FIGURE 4:**
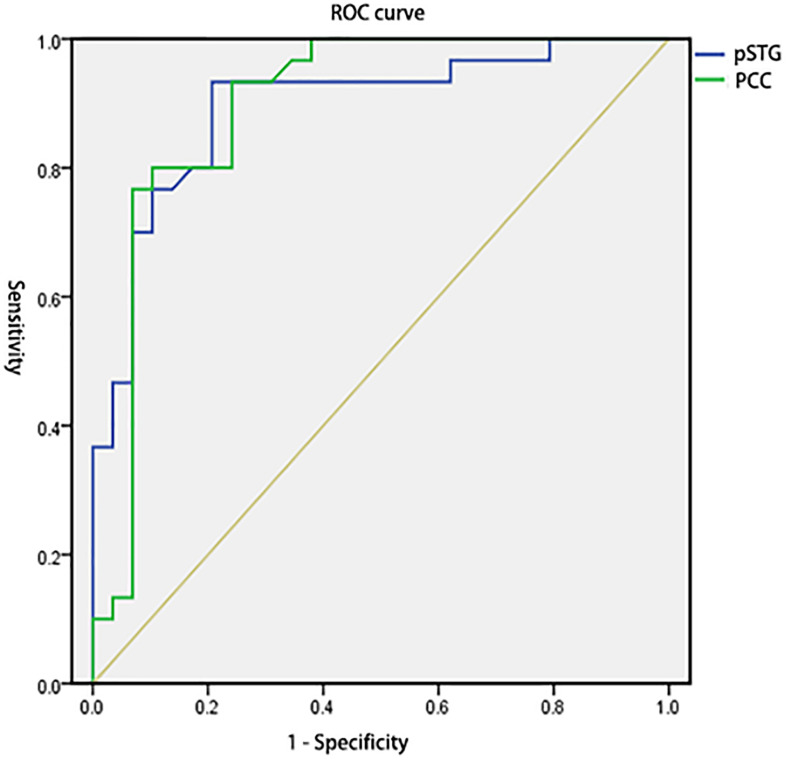
Receiver operating characteristic curves (ROC) for differentiating the amusia from the control using the VMHC values in the pSTG and PCC identified by group analysis. VMHC, voxel-mirrored homotopic connectivity; pSTG, posterior superior temporal gyrus; PCC, posterior cingulate cortex.

To test the hypothesis that whether the abnormal clusters (pSTG/PCC) can be credible neural markers to identify the amusia from the controls, we conducted the ROC analysis. The results showed that the areas under the ROC curve (AUCs) were 0.894 with pSTG (*p* < 0.001), and 0.896 with PCC (*p* < 0.001). Results also showed that the VMHC value of the pSTG could correctly classify 28 of 30 amusics and 23 of 29 controls, resulting in a sensitivity of 93.33% and a specificity of 79.31%; the VMHC value of the PCC could correctly classify 23 of 30 amusics and 28 of 29 controls, resulting in a sensitivity of 76.67% and a specificity of 96.55% (Table 3 and Figure 4).

**TABLE 3:** ROC analysis for differentiating the amusics from the controls.

Brain region	Area under the curve	Cut-off point	Sensitivity	Specificity
pSTG	0.894***	0.639^a^	93.33% (28/30)	79.31% (23/29)
PCC	0.896***	1.146^b^	76.67% (23/30)	96.55% (28/29)

ROC, receiver operating characteristic curves; VMHC, voxel-mirrored homotopic connectivity; pSTG, posterior superior temporal gyrus; PCC, posterior cingulate cortex. ^*a*^ By this cut-off point, the VMHC value of the pSTG could correctly classify amusia and control, resulting in a sensitivity of 93.33% and a specificity of 79.31%. ^*b*^By this cut-off point, the VMHC value of the PCC could correctly classify amusia and control resulted in a sensitivity of 76.67% and a specificity of 96.55%. ***P < 0.001.

## Discussion

In this study, we explored the interhemispheric FC of congenital amusia by the VMHC method. Compared to the control group, amusics showed increased VMHC in the posterior superior temporal gyrus (pSTG) and posterior cingulate cortex (PCC). Correlation analyses revealed a negative correlation between the VMHC value in pSTG/PCC and the music perception ability among amusics. ROC analyses also showed that the VMHC value of pSTG/PCC showed a good sensibility/specificity to differentiate the amusics from the controls.

The present study found increased VMHC in pSTG of amusia, which suggested the amusia had enhanced functional interactions between bilateral pSTG. The pSTG has been proposed to involve top–down feedback (IFG-pSTG) during vocal production in amusia in early life (Peretz, 2016). The DTI study of Loui et al. (2009) also suggested amusia showed abnormal connectivity between pSTG and IFG (arcuate fasciculus). Notably, our cluster is more inclined to the posterior STG extending to the temporoparietal area, which suggested enhanced FC in bilateral temporoparietal of amusia. Leveque et al. (2016) also found increased FC of the left Heschel’s gyrus with the bilateral temporoparietal area, which may also suggest functional coupling in bilateral temporoparietal in amusia. Our data also revealed increased VMHC in PCC of amusia. It has been reported no clear consensus about the function of PCC in the human brain (Raichle et al., 2001; Leech et al., 2011). One potential function of the PCC was related to awareness (Boly et al., 2008). A recent review of amusia has proposed the deficit in pitch discrimination is the visible manifestation of congenital amusia, but not the underlying functional root and the core deficit is in a lack of conscious access to processed pitch deviances (Peretz, 2016). For instance, prior ERP studies found the amusia can track and represent subtle musical pitch variation but could not make these pitch representations into a conscious report (Peretz et al., 2009; Moreau et al., 2013). Therefore, it is likely that the neural basis of conscious deficit in amusia was associated with the abnormality in PCC. Our result did not find abnormal VMHC in the auditory cortex, which reported overconnectivity between bilateral auditory cortex in prior FC (Leveque et al., 2016). Reviewing the study, it reported amusics had an overconnectivity between the left Heschl’s gyrus seed and the right auditory cortex. Notably, the right auditory cluster was located more laterally and more posteriorly in the STG than the mirror-symmetrical right Heschl’s gyrus seed. While the VMHC of the study compared each pair voxel in the symmetrical brain space, it was more sensitive to the geometric differences between the hemispheres. There also may be some demographic factors leading to inconsistent results. Firstly, the amusics were from different populations. Our study participants are from tone language background (Chinese), while the amusics from Leveque’s study were non-tone language background (French). Secondly, our participants (mean age: 19.73, age range: 18–24) are much younger than amusics from Leveque’s study (mean age: 37.08, age range: 18–57). Thirdly, our study has a relatively larger sample size (total amusics: 30, male/female: 13/17) than the prior study (total amusics: 13, male/female: 6/7). These factors have been reported affecting the resting-state FC (Dansereau et al., 2017; Gao et al., 2020; Hrybouski et al., 2021). Therefore, it is necessary to explore the resting-state FC of amusia in different language backgrounds and age groups with a large sample in the future. In total, both our results and prior FC results suggested the interhemispheric FC was abnormally increased in amusics at resting state.

Interestingly, both the pSTG and PCC in our study are overlapped with the key brain regions of the default mode network (DMN), which includes medial prefrontal cortex, PCC, and temporoparietal junctions/inferior parietal lobule^2^ (Acikalin et al., 2017). The increased VMHC in pSTG and PCC suggest amusia had increased FC within the DMN. The DMN is characterized by increased activity during passive control states as compared to most attention-demanding tasks (Greicius et al., 2003; Buckner et al., 2008). A systematic review and meta-analysis have reported that the strength of DMN FC follows an inverse U-shape with increasing age (Mak et al., 2017). The abnormality of the VMHC within DMN may reflect an immature state of the system in amusics. This is in line with prior studies, which have interpreted overconnectivity of the auditory cortex with DMN in amusia as a marker of incomplete maturation at resting state (Leveque et al., 2016; Albouy et al., 2019).

Correlation analyses revealed both the VMHC value of pSTG and PCC were negatively related to the global music score in amusics. The abnormal increased interhemispheric FC may suggest a natural deficit in amusics. One explanation was that amusia was a neurodevelopment disorder, which may be related to neurodevelopmental immaturity due to a malformation in brain development (Ayotte et al., 2002; Hyde et al., 2007). Prior findings of the abnormal corpus callosum in amusia also provide evidence for the view. For example, prior TBSS studies suggested congenital amusia (Wang et al., 2017) had higher axial diffusivity (AD) and non-recovered acquired (stroke) amusia (Sihvonen et al., 2017) had lower fractional anisotropy (FA) index in the corpus callosum, which indicted amusia lower degree of myelination in white matter tracts (Song et al., 2003; Chiang et al., 2009). It has also proposed the brain regions involved in high-order progress showed decreased homotopic FC in the normal development of the brain (Zuo et al., 2010). For instance, a prior study found the performance in visual memory tasks was negatively related to homotopic FC in the posterior portion of the brain (Gracia-Tabuenca et al., 2018). Besides, the higher performance in general intelligence was related to lower homotopic FC in sensorimotor areas (Santarnecchi et al., 2015). The overconnectivity between the bilateral hemispheres may reflect a hyper-associative condition in amusics, possibly due to a failure to properly segregate areas into distinct networks (Mitchell, 2011). The explanation is also consistent with the dedifferentiation hypothesis, which posits that bilateral activation reflects interfering recruitment of contralateral areas leading to suboptimal task performance (Baltes and Lindenberger, 1997).

Correlation analyses and ROC results also indicated the pSTG was negatively related to rhythm discrimination ability (temporal subtest/memory subtest) in amusics, and higher sensibility in amusics and related lower specificity in controls according to the VMHC value of pSTG, while the PCC was negatively related to pitch discrimination ability (melodic subtest) in amusics but higher specificity in controls and related lower sensibility in amusics according to the VMHC value of PCC. One explanation was that there were two types of amusia, which were tone deafness (pitch deficit) associated with PCC and beat deafness (rhythm deficit) associated with pSTG. Prior studies have also shown beat deafness showed deficits in beat processing and could occur in isolation with tone deafness (Phillips-Silver et al., 2011; Dalla Bella and Sowiński, 2015; Bégel et al., 2017), but most tone deafness also have rhythm discrimination ability deficit (Foxton et al., 2006; Lagrois and Peretz, 2019). For the pSTG, the sensibility/specificity may suggest the majority of individuals in the amusia group have poor rhythm discrimination ability, but some individuals in the control group may also show poor rhythm discrimination ability. For the PCC, the sensibility/specificity may suggest the majority of individuals in the control group have good pitch discrimination ability, but some beat deafness individuals in amusia may have near-normal pitch discrimination ability. Notably, both the pSTG and PCC have good diagnostic values (AUCs 0.894 for pSTG; AUCs 0.896 for PCC). It is in line with previous classification analysis in neuroimaging data of congenital amusia, which reported that resting-state connectivity data was able to discriminate amusia from typical controls (Albouy et al., 2019). Our results also provided evidence for the view of Zuo et al. (2010), who proposed the VMHC approach could be used to facilitate the discovery of interhemispheric interaction in the development disorders. These results also suggest the overconnectivity within DMN (pSTG/PCC) may be a credible neural marker to classify amusics from control individuals. It would be necessary to estimate the power of the VMHC classification in identifying congenital amusia in the future.

The present study has some limitations. Firstly, gray/white matter abnormalities were not assessed, which has been reported to be associated with the VMHC deficits in psychiatric disorders such as schizophrenia (White et al., 2011). Therefore, there is no way to know the potential influence in the VMHC of amusia in this study. Secondly, though it has proposed the correlation of the fMRI bold signals of gray matter is considered to be an indirect index of synchrony in the spontaneous neural activity of the brain and showed interhemispherical coherent fluctuations of cortical neurons signals in animals (Fox and Raichle, 2007; Innocenti, 2009; Biswal et al., 2010), further work is needed to confirm the VMHC approach to predict behavioral performance in other developmental and learning disorders such as dyslexia and prosopagnosia in resting-state fMRI data. Thirdly, a recent study found the hemispheric asymmetry of FC and the intrahemispheric FC were both related to visual memory and visual attention tasks in school-age children (Gracia-Tabuenca et al., 2018), which may also influence adult amusic individuals in the auditory domain. Therefore, the hemispheric specialization of the resting-state needs to explore in the future for a better understanding of the relationship between the two hemispheres of amusia.

## Conclusion

In summary, the present study was firstly, directly to explore the interhemispheric functional interaction characteristics in congenital amusia. Results indicated amusics showed abnormally increased VMHC within the posterior part of the DMN mainly in the posterior superior temporal gyrus (pSTG) and PCC. Correlation analyses among amusics revealed a negative correlation between the VMHC value in pSTG/PCC and the music perception ability. Further ROC analyses showed that the VMHC has a good sensibility/specificity to differentiate the amusics from the controls. These findings provide a new perspective for understanding the neural basis of congenital amusia and imply the immature state of DMN in amusia may be a credible neural marker.

## Data Availability Statement

The original contributions presented in the study are included in the article/Supplementary Material, further inquiries can be directed to the corresponding author/s.

## Ethics Statement

The studies involving human participants were reviewed and approved by the ethics committee of the Second Xiangya Hospital of Central South University. The patients/participants provided their written informed consent to participate in this study.

## Author Contributions

DW and ZJ designed the study. ZJ, SH, LJ, YY, MX, JW, and QL performed the research. ZJ analyzed the data. ZJ and DW wrote the manuscript. All authors contributed to the article and approved the submitted version.

## Conflict of Interest

The authors declare that the research was conducted in the absence of any commercial or financial relationships that could be construed as a potential conflict of interest.

## Footnotes

1 http://rfmri.org/dpabi

2 https://identifiers.org/neurovault.collection:1653

## Supplementary Material

Supplementary Figure 1 Correlation analyses between the mean VMHC value of pSTG and MBEA scores in control group.

Supplementary Figure 2 Correlation analyses between the mean value of PCC and MBEA scores in control group.
