# Supplementary material for: Increased Resting-State Interhemispheric Functional Connectivity of Posterior Superior Temporal Gyrus and Posterior Cingulate Cortex in Congenital Amusia
Source: Front Neurosci. 2021 Apr 30;15:653325. doi: 10.3389/fnins.2021.653325 (PMC8120159; doi:10.3389/fnins.2021.653325)
Supplement: Supplementary Figure 1 — Correlation analyses between the mean VMHC value of pSTG and MBEA scores in control group. [file Image_1.pdf]

Supplementary material

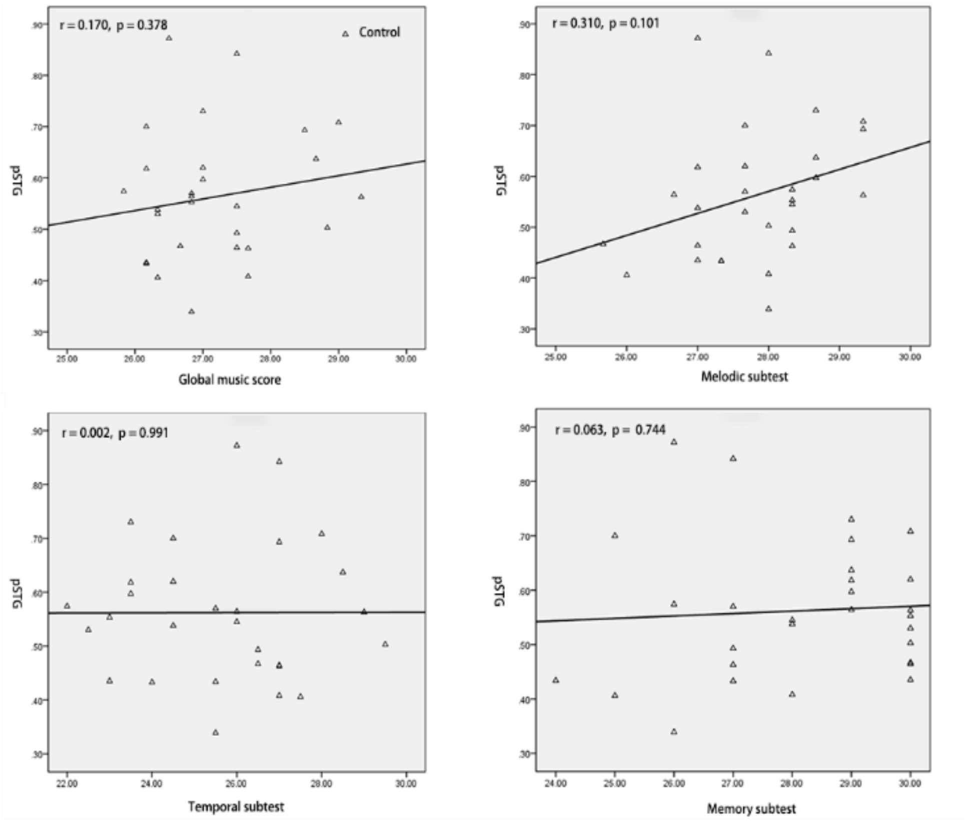

Figure S1. Correlation analyses between the mean VMHC value of pSTG and MBEA scores in control group.

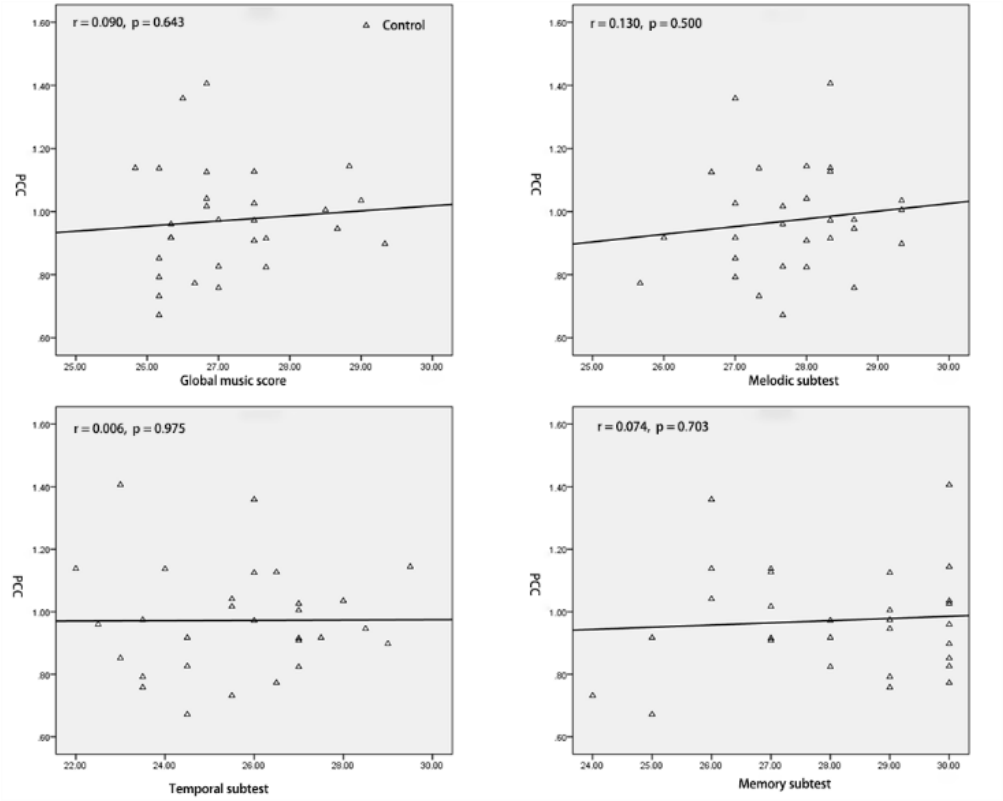

Figure S2. Correlation analyses between the mean value of PCC and MBEA scores in control group.
